# Implementing healthy food environment policies in New Zealand: nine years of inaction

**DOI:** 10.1186/s12961-021-00809-8

**Published:** 2022-01-15

**Authors:** Sally Mackay, Sarah Gerritsen, Fiona Sing, Stefanie Vandevijvere, Boyd Swinburn

**Affiliations:** 1 Department of Epidemiology and Biostatistics, School of Population Health, University of Auckland, Auckland, 1023 New Zealand; 2 Centre for Longitudinal Research He Ara Ki Mua, School of Population Health, University of Auckland, Auckland, 1743 New Zealand; 3 Sciensano (Scientific Institute of Public Health), Epidemiology and Public Health, J.Wytsmanstraat 14, 1050 Brussels, Belgium

**Keywords:** Food environments, Government policy, Nutrition, Accountability, INFORMAS, Obesity prevention

## Abstract

**Background:**

The INFORMAS [International Network for Food and Obesity/Non-communicable Diseases (NCDs) Research, Monitoring and Action Support] Healthy Food Environment Policy Index (Food-EPI) was developed to evaluate the degree of implementation of widely recommended food environment policies by national governments against international best practice, and has been applied in New Zealand in 2014, 2017 and 2020. This paper outlines the 2020 Food-EPI process and compares policy implementation and recommendations with the 2014 and 2017 Food-EPI.

**Methods:**

In March–April 2020, a national panel of over 50 public health experts participated in Food-EPI. Experts rated the extent of implementation of 47 “good practice” policy and infrastructure support indicators compared to international best practice, using an extensive evidence document verified by government officials. Experts then proposed and prioritized concrete actions needed to address the critical implementation gaps identified. Progress on policy implementation and recommendations made over the three Food-EPIs was compared.

**Results:**

In 2020, 60% of the indicators were rated as having “low” or “very little, if any” implementation compared to international benchmarks: less progress than 2017 (47%) and similar to 2014 (61%). Of the nine priority actions proposed in 2014, there was only noticeable action on one (Health Star Ratings). The majority of actions were therefore proposed again in 2017 and 2020. In 2020 the proposed actions were broader, reflecting the need for multisectoral action to improve the food environment, and the need for a mandatory approach in all policy areas.

**Conclusions:**

There has been little to no progress in the past three terms of government (9 years) on the implementation of policies and infrastructure support for healthy food environments, with implementation overall regressing between 2017 and 2020. The proposed actions in 2020 have reflected a growing movement to locate nutrition within the wider context of planetary health and with recognition of the social determinants of health and nutrition, resulting in recommendations that will require the involvement of many government entities to overcome the existing policy inertia. The increase in food insecurity due to COVID-19 lockdowns may provide the impetus to stimulate action on food polices.

**Supplementary Information:**

The online version contains supplementary material available at 10.1186/s12961-021-00809-8.

## Background

New Zealand’s food environments are characterized by highly accessible and heavily promoted energy-dense, often nutrient-poor, food and drinks that contain high levels of salt, saturated fats and sugars [1, 2]. Food environments are major drivers of unhealthy diets and energy overconsumption [3–5]. Collectively, unhealthy diets are the greatest contributor to the preventable health burden in New Zealand. High body mass index (BMI) contributes 8.3% and other dietary risks (such as high salt intake, low fruit and vegetable intake) contribute 8.6% of disability-adjusted life-years (DALYs) lost [6]. Combined, this is greater than the estimated 9.7% of health loss from tobacco use [6, 7].

New Zealand adults have the third highest rate of obesity [8] and children the second highest prevalence of obesity [9] within Organisation for Economic Co-operation and Development (OECD) and European Union (EU) countries. In 2018 and 2019, 31% of adults had obesity, up from 27% in 2006/2007, and one in nine children aged 2–14 years (11%) had obesity [10]. Adult and child obesity rates were higher for Māori and Pacific Peoples and for those living in areas of high deprivation [10].

Effective government policies and actions across settings and sectors are essential to increase the healthiness of food environments and to reduce obesity, diet-related noncommunicable diseases (NCDs) and their related inequities [11]. Food policies need to align to achieve common health, environmental, social and economic goals, to improve the overall food system without undermining one part of it [12]. Internationally, some governments have demonstrated leadership and taken action to improve the healthiness of food environments. These can serve as best practice exemplars or benchmarks for other countries. Despite wide recognition of obesity and diet-related NCDs as a major public health issue internationally, the New Zealand government has been slow to improve food environments. This is in part due to the pressure of the food industry on governments [13, 14] and other factors such as the challenges of providing robust evidence in emerging policy areas and the competition for resources between prevention efforts and health services delivery [15, 16]. Non-cohesive, diverse requests from public health advocates to address unhealthy food environments are unhelpful [16], and so an agreed prioritization of policy demands serves as an effective tool when lobbying for change.

The International Network for Food and Obesity/NCDs Research, Monitoring and Action Support (INFORMAS) [3] developed a tool and process, the Healthy Food Environment Policy Index (Food-EPI) [17], to assess the level of implementation of government policies and infrastructure support compared to international best practice for improving food environments and population diets. The Food-EPI tool and process have been through several phases of development, pilot-tested in New Zealand in 2014 [18, 19], and since implemented (or in progress) in 40 low-, middle- and high-income countries. New Zealand is the first country to implement the tool three times, aligned to political electoral cycles in order to stimulate debate.

This paper presents the results of the third Food-EPI study in New Zealand and compares the government’s progress on policy and infrastructure support for healthy food environments in 2020 with 2017 and 2014. We also compare the priority actions recommended by experts in 2020 with priorities in 2017 and 2014.

## Methods

The Food-EPI comprises a “policy” component with seven domains on specific aspects of food environments and an “infrastructure support” component with six domains to strengthen obesity and NCD prevention systems. Good practice indicators contained in these domains encompass policies and infrastructure support necessary to improve the healthiness of food environments and to help prevent obesity and diet-related NCDs. The overview and principles of the development of the methods has been described previously [3] and is summarized in Additional file 1. Food-EPI indicators are consistent with proposed international policy options [20–22]. Food-EPI aims to create a common understanding between public health experts to advocate governments on the priorities for policy action.

A mixed-methods design was used to obtain the ratings of the level of implementation of good practice policies and infrastructure support, and to identify and prioritize actions to fill implementation gaps.

### Expert panel

Public health experts from a wide range of organizations were invited to take part in the Food-EPI as part of an expert panel to ensure expertise for all aspects of policy implementation. Experts invited were academics, researchers and practitioners, and those working in public health nongovernmental organizations (including medical associations, professional bodies and service providers) were invited to take part in the Food-EPI. These included participants from the 2014 and 2017 expert panels. If an expert was not able to participate, they were asked to invite a colleague to participate in their place. Government experts (e.g. from different ministries, the Health Promotion Agency and district health boards) were also invited to participate. All participants on the expert panel provided informed consent before taking part in the appraisal. Government experts, acting as observers, were present to provide clarification or additional information but did not participate in the ranking of actions. This was also the case in 2017, but government experts were not part of the expert panel in 2014.

### International best practice exemplars (benchmarks)

Benchmarks were selected for each of the good practice indicators from the World Cancer Research Fund International NOURISHING framework [22] and obtained from international food policy experts. Some examples of benchmark policies are the front-of-pack warning labelling system in Chile, the regulatory norms defining limits for foods high in certain nutrients in Chile, the sugar industry levy on sugar-sweetened beverages in the United Kingdom, the inclusion of cultural, ethical and environmental perspectives in the Brazilian dietary guidelines, and the nutrient profiling system used to prevent unhealthy food products carrying health claims in Australia and New Zealand. The full list of benchmarks is available in Additional file 2.

### Evidence compilation and verification

For each Food-EPI (2014, 2017, 2020), an evidence document was compiled outlining the current extent of implementation of all 47 good practice policy and infrastructure support indicators (43 in 2014) across 13 domains, as outlined previously [18], for the expert panel to carry out their assessment [23]. Information was compiled from policy documents, websites and budgets retrieved from websites and through Official Information Act requests and personal communication with government officials. The evidence was comprehensively documented and returned to government officials to verify its completeness and accuracy.

### Rating implementation progress

The expert panel rated the level of implementation in New Zealand against each good practice indicator using the evidence document for reference. This was conducted in February and March 2020 using an anonymous online survey (Qualtrics) ahead of the workshop. Each expert gave a rating for each indicator on a Likert scale of 1 to 5. A rating of 1 meant the expert panel member believed the New Zealand government had implemented the indicator between 0 and 20% compared to international best practice, and a rating of 5 indicated implementation of between 80 and 100% compared to best practice. These were compared to the results of the 2017 and 2014 Food-EPI assessments. The 2017 rating process was carried out using an online survey in April and May, while in 2014, two workshops were convened to obtain ratings. This process was changed after 2014 after receiving evaluative feedback from the 2014 expert panel and learning from other Food-EPI processes that had taken place internationally.

### Action and prioritization workshops

At the workshops, the expert panel met to collectively identify the actions required and prioritize these according to their importance and achievability. In 2020, the implementation of the workshops was affected by COVID-19 restrictions on travel and social distancing. One face-to-face workshop was held in Auckland (19 March) and one online workshop was held via Zoom (8 April) to replace the planned face-to-face workshops in Wellington and the South Island. At the face-to-face workshop, participants decided whether an action was required for an indicator, then reviewed the 2017 action and decided whether to keep the 2017 action, revise it or a develop a new action. Due to time restrictions on the part of public health experts during the COVID-19 pandemic, the actions developed at the Auckland face-to-face workshop were presented to participants in the online workshop. Participants discussed the high-priority actions verbally or via the chat feature and revised the action or developed a new action. The action was displayed in the chat feature and a vote was taken to assess whether the majority of experts were in favour.

During the workshops, the proposed actions were identified as higher or lower priority. Following the workshops, the higher-priority actions were ranked by participants from both workshops using an online survey (Qualtrics) sent to all expert panel members a week after the online workshop. Participants were asked to separately prioritize the importance and achievability of each action, for policies and infrastructure support separately. Importance was defined as the relative need, impact, effects on equity, and any other positive or negative effects of the action. Achievability was defined as the relative feasibility, acceptability, affordability and efficiency of the action. Participants were asked to consider “acceptability to government” as pertaining to New Zealand governments in general, not the government of the day.

The results of the 2017 and 2014 Food-EPIs have been reported previously [18, 24].

### Data analysis

The mean rating for each indicator was used to determine an overall percentage level of implementation. These ratings were then categorized into “high”, “medium”, “low” or “very little, if any” levels of implementation based on the following cut points: > 75% = high; 51–75% = medium; 26–50% = low; ≤ 25% = very little, if any.

For the prioritization of actions, graphs were created to plot importance against achievability. In general, actions rated highest for both importance and achievability were selected as top priorities. A bar graph was created to compare the level of implementation of the indicators between 2014, 2017 and 2020. The content of the actions prioritized by the expert panel was compared between 2014, 2017 and 2020.

## Results

### Expert panel

Participation in the 2020 expert panel was lower than in previous years due to the COVID-19 pandemic, with 27 participants completing the online rating. Ten participants attended the face-to-face workshop. The videoconference workshop was attended by 25 independent participants and four government observers. Thirty-one of the 35 workshop participants (independent experts) completed the online ranking of actions (89% response rate). A total of 39 actions were proposed, 22 as higher priority (and subsequently ranked by experts) and 17 as lower priority. Some actions covered more than one indicator, such as the proposed action to develop a long-term, multisectoral national food systems and nutrition strategy.

### Ratings and progress

**Fig. 1:**
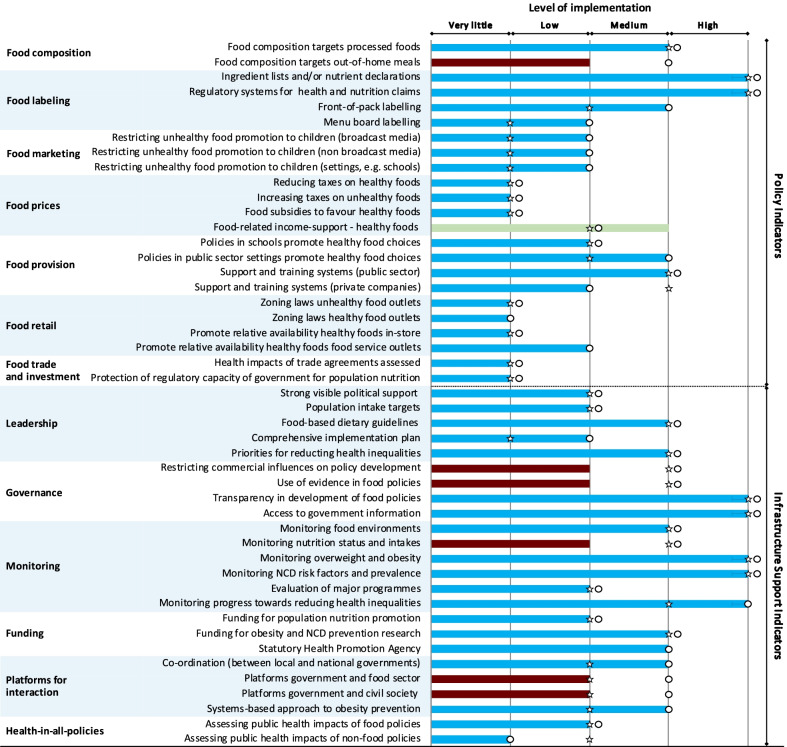
Level of implementation of food environment policies and infrastructure support by the New Zealand Government in 2020 against international best practice. Star: 2014 Food-EPI ratings; circle: 2017 Food-EPI ratings; Change in level of implementation: Brown: Reduced since 2017; Blue: No change since 2017; Light green: Progress since 2017

Figure 1 presents the level of implementation as rated by the expert panel over the three time points. In 2020, three fifths (59.5%) of all the indicators were rated as having “low” or “very little, if any” implementation compared with international benchmarks (49.0% in 2017 and 60.5% in 2014). In 2020, 15% of indicators were rated as high implementation, which was similar to 2014 and 2017 (14%, 15%). In 2020, two thirds (69.5%) of the policy indicators and half (50%) of the infrastructure indicators were rated as “low” or “very little, if any” implementation. This was similar to 2014 (75% policy, 48% infrastructure) and to 2017 for policy (70%) but different for infrastructure in 2017, which had dipped to a low of 29% of indicators ranked as low” or “very little, if any” implementation.

Between 2014 and 2020 for the 43 indicators available for each time period, 26 indicators (60%) received the same implementation ranking over all three time periods, 11 indicators had an increase in level of implementation and six indicators had a lower rate, with almost all of the progress occurring between 2014 and 2017.

New Zealand has been rated consistently well against international best practice for six indicators, as indicated in Fig. 1. Two relate to food labelling indicators in the policy section and four relate to different infrastructure support indicators: transparency in the development of food policies; public access to nutrition information; regular monitoring of NCD risk factors and health-related inequalities.

There were 20 indicators for which New Zealand was rated consistently poorly against international best practice (low, very little, if any implementation). Most of these were policy indicators (14, 70%), including implementing restrictions on unhealthy food marketing to children; healthy food policies in schools; fiscal policies to support healthy food choices; limiting the density of unhealthy food outlets; food composition targets/standards in out-of-home settings; and ensuring that trade and investment agreements do not negatively affect population nutrition and health. The six infrastructure indicators were related to leadership, evaluation of major programmes, funding for population nutrition promotion and assessing public impacts of food and non-food policies.

The indicators where implementation levels improved over the period 2012 to 2020 were related to the introduction in 2017 of the Advertising Standards Authority (ASA) self-regulatory code restricting marketing of unhealthy food and beverages to children; the Health Star Rating front-of-pack labelling programme in 2014; the introduction of the National Healthy Food and Drink Policy in 2016 for district health boards and government agencies; and the introduction of the Childhood Obesity Plan in 2015. However, the ASA self-regulatory system has been evaluated as ineffective [25], and the Childhood Obesity Plan has not been widely implemented.

The indicators where implementation regressed since 2017 were the regular monitoring of adult and childhood nutrition status and population intake; food composition targets for out-of-home meals; restricting commercial influences on policy development (this regressed as the government strengthened engagement platforms with industry, for example industry pledges as part of the Healthy Kids Industry Pledge); and formalizing a platform for civil society participation in improving food environments.

### Actions and priorities

**Fig. 2:**
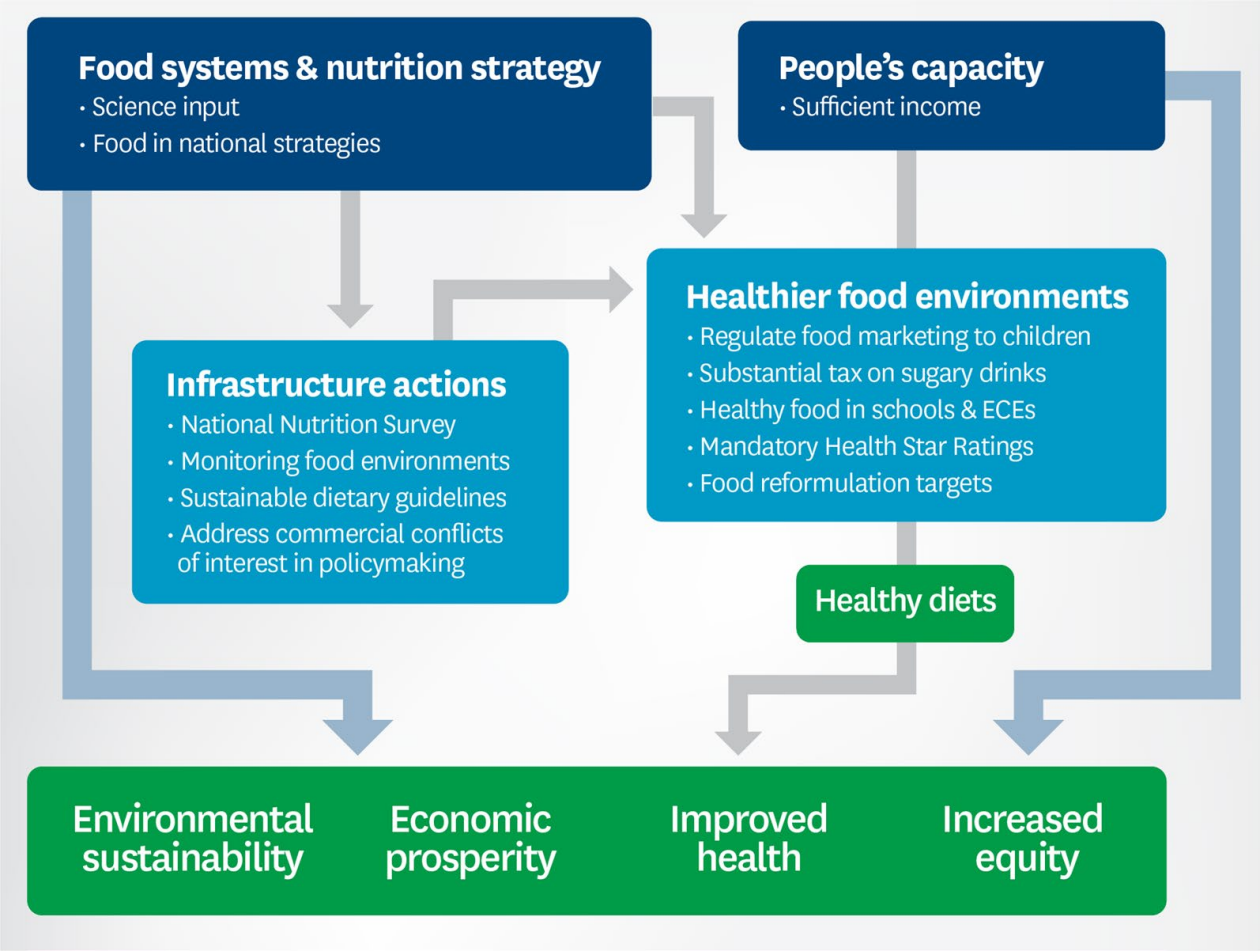
Recommendations from the expert panel prioritized for immediate action to improve food environments in 2020

In 2020, of the 39 actions proposed during the workshops (Additional file 2), eight policy actions and 14 infrastructure support actions were considered of high priority. Some actions covered more than one indicator. The expert panel prioritized 13 for immediate action (Fig. 2) in terms of feasibility and achievability.

The prioritized actions were compared across 2020 and the previous years (reported in previous publications [18, 24]) (Table 1) with the key theme of the action indicated in italics. Some of the actions were almost identical over the three time periods: restricting marketing to children; food composition targets for sodium and added sugar; and a sugary drinks levy. The action to ensure that food provided in or sold by schools and early childhood education services met dietary guidelines had a similar theme across years, with the addition of the need for a food policy in 2020.

**Table 1:** Prioritized recommendations of expert panels from 2014, 2017 and 2020 to improve the food environment

2014	2017	2020
*Implement a comprehensive national action plan* for obesity and NCD prevention	*Strengthen the Childhood Obesity Plan* including policy objectives and targets to reduce obesity prevalence and inequalities, and more and stronger policies to create healthy children’s food environments and increasing funding for the implementation and evaluation of the plan	The government *develops a long-term, multisectoral national food systems and nutrition strategy* with clear outcomes and indicators to improve sustainability, food sovereignty, health and equity, and to honour the rights of Māori to the protection of their health under the Te Tiriti o Waitangi^a^ The government appoints a *food and nutrition scientific committee* to work with the ministerial science advisors to ensure policies related to food and nutrition are evidence-based and equitable The government ensures that the implementation plans for existing, relevant government actions, such as the Child and Youth Wellbeing Strategy, include priorities to improve food environments, beyond the Healthy Active Learning
		The government *supports low-income households* so they can afford a healthy diet
*Set priorities in statements of intent and set targets for*: Reducing childhood and adolescent obesity Reducing salt, sugar and saturated fat intake Food composition (salt and saturated fat) in key food groups	*Set targets for*: Reducing childhood overweight and obesity by 8 percentage points (from one third to one quarter) by 2025 with decreasing inequalities Reducing mean population intake of salt, sugar and saturated fat based on WHO recommendations Voluntary reformulation of composition (salt, sugar and saturated fat) in key food groups	The government adopts a two-tier system for reducing sodium and added sugar in key food categories: setting *mandatory maximum levels* that reduce over time, and setting and monitoring targets for voluntary reductions in sales-weighted averages
*Increase funding* for population nutrition promotion, doubling it to at least $70 million/year	*Increase funding* for population nutrition promotion to at least 10% of obesity/overweight healthcare costs	
*Reduce the promotion of unhealthy foods to children and adolescents by:* **Restricting the marketing of unhealthy foods to children and adolescents through broadcast and non-broadcast media** Ensuring that schools and early childhood education services are free of commercial promotion of unhealthy foods	*Regulate unhealthy food marketing, as defined by the WHO nutrient profiling model, to children up to 18 years* In broadcast media, including during children’s peak viewing times (e.g. up to 9 pm) In non-broadcast media, including food packaging, sports sponsorship and social media In children’s settings, including “school food zones”	The government introduces *regulations to restrict unhealthy food and beverage marketing to children up to 18 years old* through broadcast media (during peak TV viewing times), non-broadcast media (including food packaging, sport sponsorship and digital media) and in children's settings, using the WHO nutrient profiling models, tailored to the New Zealand context
*Ensure that food provided in or sold by schools and early childhood education services* meets dietary guidelines	*Ensure healthy foods in schools and early childhood education services* using the updated Ministry of Health Food and Beverage Classification System	The government *requires and supports schools and early childhood education services to develop food policies* which ensure healthy foods are provided and promoted
***Implement the front-of-pack Health Star Rating* labelling system**	***Strengthen the Health Star Rating system* by urgently addressing anomalies in the design algorithm (especially for sugar), increasing funding for promotion and making it mandatory if there is not widespread uptake by 2019**	The government makes the *Health Star Rating mandatory* and adopts the recommendations on changes to the algorithm and beverages of the 5-year review of the Health Star Rating system
*Introduce an excise tax* of at least 20% on sugar-sweetened beverages	*Introduce a substantial (e.g. 20%) tax on sugar-sweetened beverages* and explore using the revenue for programmes to improve public health and well-being	The government *introduces a tiered industry levy of at least 20% on sugary drinks* and recycles the revenue for programmes to improve public health and well-being
	*Implement the new Eating and Activity Guidelines* by increasing funding for their promotion and translating them for New Zealand’s social, environmental and cultural contexts	The government *actively implements and increases funding to promote Eating and Activity Guidelines* which incorporate the social, environmental and cultural dimensions of eating
	*Conduct a new national nutrition survey for children* within 3 years and institute a plan for future regular adult and children nutrition surveys	The government conducts a *new national nutrition survey for children and adults* to be commissioned by 2021 The government *regularly monitors the food environment* for health, equity and sustainability
		The government *expands its conflict of interest procedures to include commercial conflicts and transparency measures* so that consultation with the food industry can continue without it exerting undue influence on government policy development

Bolded text indicates recommendations for which the government has made progress

^a^Te Tiriti o Waitangi is New Zealand’s founding document. It is an agreement made between representatives of the British Crown and Māori Rangatira (chiefs)

A few actions proposed by the expert panels changed over time, mostly due to some implementation of the original proposed action. An action plan/strategy was recommended at each Food-EPI, starting with an obesity and NCD prevention plan in 2014. The introduction of the Childhood Obesity Plan in 2015 was reflected in the 2017 recommendation to strengthen this plan. However, this plan was effectively ignored by the next government. The 2020 action was instead multisectoral in nature, recommending a food systems and nutrition strategy. The government entities with a role in food policy were identified (Table 2). An action related to the Health Star Rating labelling system was prioritized each year. In 2014, this was to implement the Health Star Rating, which occurred in 2014, so in 2017 the action was related to improving the algorithm and mandatory implementation. A review of the Health Star Rating algorithm took place in 2019, so the 2020 action was related to making the Health Star Rating mandatory and implementing the review recommendations.

**Table 2:** Description of government entities with a role in food policy

Government departments	Descriptions
Ministry of Health	Main policy-making department on diet-related health, nutrition-related health inequalities, planning and funding public health and monitoring the performance of district health boards
Ministry for Primary Industries	Main policy-making department for New Zealand's primary industries, including food. Functions include providing national direction on ensuring the food produced is safe, enabling international market access for New Zealand’s primary products, and representing the interests of the New Zealand primary sector in international trade policy and standard-setting forums
Ministry of Foreign Affairs and Trade	Main policy-making department on international food trade, overseas aid (including food aid), overseas agriculture, and the Sustainable Development Goals
Health Promotion Agency	Main communications agency to promote health, including healthy diets
Ministry for the Environment	Main policy-making department on environmental policy and provides national direction on urban (e.g. food density zoning laws) and rural planning (e.g. land use consents) through national policy statements and national environmental standards. Also focuses on climate change, fresh water, marine, land, waste, soil, air, water, sea quality
Ministry of Business, Innovation and Employment	Main policy-making department managing food and beverage industry investment, consumer protection, immigration (including migrant workers for food supply chain), business, industrial strategy, employment, energy, science, research and innovation (all with food relevance)
Food Standards Australia New Zealand	Develops and administers joint Australia and New Zealand food standards; explains food issues e.g. labelling, additives, chemicals; consults with the community about food safety issues; helps food businesses understand the Australia New Zealand Food Standards Code
Ministry of Education	Main policy-making department on education, skills and curriculum, with role as food educator and food provider
Office of the Prime Minister’s Chief Science Advisor	Provides strategic advice across sectors and serves as an accessible conduit between the science community and government
Local government	Ensures public services are responsive to the social, economic, environmental and cultural well-being needs of their communities, with a particular role in zoning law, district or regional planning, and community food supply initiatives for example
District health boards	A role to improve, promote and protect the health of people and communities, including planning and delivering services in their area
The Treasury	Overall control of government spending
Department of Prime Minister and Cabinet	Overall policy oversight and coordination. Contains the Child Wellbeing and Poverty Reduction Group
Te Puni Kōkiri—Ministry of Māori Development	Input into major food policies as they relate to Māori
Ministry for Pacific Peoples	Input into major food policies as they relate to Pacific Peoples
Ministry of Social Development	Main policy-making department on welfare and pensions, supporting people and whānau in food poverty
Supporting government entities	
Health Research Council of New Zealand	Sets priorities for research and funds research including on food and nutrition
Broadcasting Standards Authority	Decides complaints about broadcasters; publishing and research broadcasting standards
Sport New Zealand	Oversees sports sponsorship
Commerce Commission	Enforces laws that promote competition and protect consumers in New Zealand
National Ethics Advisory Committee	Provides ethical advice on issues of national significance in respect of health and disability, including characteristics of a fair food system delivering nutritional outcomes
Crown Research Institutes	AgResearch: pastoral, agri-food and agri-technology sector Plant and Food Research: horticultural, arable, seafood, and food and beverage industries Institute of Environmental Science Research: safeguards people's health, protects the food-based economy, improves the safety of water resources
Health and Disability Commissioner	Works with clinicians, providers and consumers to improve health services including dietary advice and interventions
Office of the Children’s Commissioner	Advocates for the interests of young people, ensuring the voices of children are heard in policy-making
Ministry for Culture and Heritage	Funds Broadcasting Standards Authority, NZ On Air and Sport New Zealand
Ministry of Transport	Main policy-making department on transport, with role in supporting infrastructure for food distribution and public transport (including for food workers and customers)
Department of Corrections	Main department with role as food provider to prisons
Department of Internal Affairs	Conduit for local and central government
State Services Commission	Sets standards for public servants and policy-making, including the management of conflicts of interest for food policies
New Zealand Customs Service	Provides border control and protects the community from potential risks related to food arising from international trade and travel, as well as collecting duties and taxes on imports to the country
Ministry of Housing and Urban Development	Main policy-making department on housing, built environment and urban development

Two new actions were introduced in 2017 and one in 2020. In 2017 and 2020 the expert panel recommended actions to implement the Eating and Activity Guidelines introduced in 2015, and to conduct a national nutrition survey (2017, a children’s nutrition survey; 2020 a children’s and adult nutrition survey). In 2020, the expert panel introduced the importance of ensuring that households have sufficient income as a high-priority action, and an action on conflict of interest procedures when consulting with the food industry.

Some actions were proposed but not prioritized in all years, despite the action not being implemented. In 2014 and 2017, actions related to setting targets to reduce childhood obesity and population intake of salt, sugar and saturated fat were prioritized, but were not considered priority actions in 2020. Increased funding for population nutrition promotion was recognized as an action for each year, but only prioritized for 2014 and 2017.

## Discussion

The 2020 Food-EPI study assessed the New Zealand Government’s progress towards international best practice in improving food environments and implementing obesity and diet-related NCD prevention policies, and compared this with earlier similar assessments in 2017 and 2014, finding little or regressed progress over this time period.

### Implementation

The results indicate that overall, almost no progress has been made since the last Food-EPI assessments in 2017 and 2014, and New Zealand has not increased its performance compared with international best practice. For those indicators that had changed since the 2017 assessment, the majority had decreased in levels of implementation (six) with only one area rated as having progressed since 2017.

There was some improvement in the level of implementation due to the introduction of some policies and interventions; however, experts recommended further actions, as implementation has not been sufficient to improve food environments and population diet. There has been no statistically significant change in the prevalence of overweight and obesity in adults or in children during the time period covered by the Food-EPI assessments (2012–2020) [10].

### Actions

Reflecting on the changes (or lack of change) over time, the actions proposed in 2014 continued to be high-priority items in 2017 and 2020. The only action which has seen progress over time is the Health Star Rating front-of-pack labelling, with a 5-year review and changes to the algorithm [26], and even with this, a mandatory programme has not been implemented as recommended by experts.

Compared to earlier years, the 2020 actions reflect a growing movement to locate nutrition within the wider context of planetary health, with recognition of the social determinants of health and nutrition, resulting in higher-level actions proposed that will require the involvement of many government entities. Connecting obesity with climate change and food security will aid progress for all [27]. The expert panel in 2020 was adamant that there needs to be clear leadership and the development of a multisectoral national food systems and nutrition strategy that honours the rights of Māori (New Zealand’s indigenous population) under Te Tiriti o Waitangi (New Zealand’s founding document) guided by a scientific committee. This recommendation echoes calls from other experts [28] and groups, such as the Food Systems Dialogues [29], Child Poverty Action Group [30] and Eat New Zealand [31], for an overarching strategy, prompted by the United Kingdom Government announcing the establishment of their National Food Strategy in 2020 [32].

The experts expressed concern about the extent of food insecurity in the country and widening health inequities, prioritizing the policy action of ensuring that households receive an adequate income to enable autonomy to make healthy food choices. One in five children live in households experiencing moderate to severe food insecurity [33], and concern about this issue has grown during the COVID-19 crisis [34]. The Child Poverty Reduction Act 2018 [35] requires monitoring of some of the underlying determinants of health, but for substantial change to occur, the Welfare Expert Advisory Group’s recommendations require implementation [36]. The disruption of food environments [37], increase in food insecurity due to COVID-19 lockdowns [38] and shift towards an unhealthy dietary pattern [39] may provide the impetus to stimulate action on food polices.

Of continued and growing concern among the Food-EPI expert panel, along with other organizations [40, 41], was the need for another national nutrition survey. Major policy decisions are being made in the absence of evidence about the nutrition status and food consumption patterns of the population. The COVID-19 crisis illustrated the importance of using epidemiological evidence as a foundation for a public health response; this applies equally to the chronic crisis of obesity and unhealthy diets.

The expert panel called for a mandatory approach to be adopted in all policy areas prioritized in 2020, as current voluntary approaches have proven to be ineffective for marketing of unhealthy food to children, Health Star Ratings labelling, healthy food policies in schools and early learning services. Voluntary policies are not enforceable and therefore not implemented or adhered to [42]. Strong government policy is essential to achieve an equitable and sustainable food system [43]. For example, only 23% of products displayed a Health Star Rating in 2019 [44], and the School Food Environment Review and Support Tool (School-FERST) study found that only 38.5% of primary schools and 44.8% of secondary schools had a healthy food policy, with most assessed to be low in strength and comprehensiveness [45].

### Implications

Despite providing the government with direction on the recommended actions to remedy areas where New Zealand’s performance is falling short through previous Food-EPI, minimal progress has been made. In the years contributing to the 2014 and 2017 Food-EPI assessments, New Zealand was governed by a centre-right minority government, who were replaced in 2017 by a centre-left coalition government. Expectations that a more left-leaning government would implement policies to improve food environments were not met. Driving this policy inertia are three main factors: inadequate political leadership and governance to enact policies; strong opposition to such policies by powerful commercial interests; and a lack of public demand for policy action [46]. Further investigation is needed to examine the surrounding determinants of the lack of action for particular indicators, to move towards overcoming this policy inertia. While Food-EPI has stimulated little progress in New Zealand, without independent expert panels measuring the government’s performance and comparing it over time, there would be little evidence on which to base calls for policy change and to measure the degree of policy inertia. Progress on recommended actions has occurred in other countries where Food-EPI was undertaken, such as the Australian Government’s agreement to the development of a national strategy on obesity [47], a sugar levy introduced in the United Kingdom [48, 49] and legislation in Mexico for front-of-pack warning labels [50, 51].

Food-EPI assessed national-level policies and infrastructure action, but future assessments could include local government and district health boards, as they too play a significant role by implementing unique food environment policies at the local level of jurisdiction, such as zoning laws for marketing or incentives to food outlets selling healthier foods. In Canada, “local Food-EPIs” have been successfully conducted in three municipal jurisdictions [52–54]. A separate study benchmarked the commitments of the major food companies in New Zealand related to population nutrition and obesity prevention [55].

The Food-EPI expert panel represents a wide range of organizations from academia, public health units, government policy-makers, nongovernmental organizations and professional organizations. A particular strength of the study is that the evidence document is verified by government officials to ensure it is correct and up to date. Food-EPI has now been completed three times in New Zealand and completed (or in progress) in 40 countries globally, and is therefore a tested and accepted tool for monitoring government progress on improving food environments.

A limitation of the 2020 Food-EPI was that it coincided with the COVID-19 pandemic, which meant many public health experts had limited, if any, time to participate. Despite this, the participating experts were fully engaged and made a valuable contribution. Two changes made to the workshop proved beneficial and are recommended for future Food-EPI. First, having the option of a video teleconference enabled more experts to participate. Second, shifting the prioritization of selected actions to an online survey after the workshops allowed time for reflection and was completed by almost all workshop participants. The Food-EPI tool does not directly capture wider policy action that may address the underlying determinants of health, such as sufficient income to enable healthy food choices, as this is broader than the indicators in the food prices domain which related to food subsidies and taxes rather than income. This research could be complemented by research that investigates public opinion of the proposed policy recommendations with recommendations for other policy actions.

## Conclusions

There has been virtually no progress in New Zealand over the past decade on the implementation of policies and infrastructure support for healthy food environments, with overall regression seen between 2017 and 2020. While there are some areas where New Zealand is at the level of best practice, almost two thirds of the Food-EPI indicators show major implementation gaps that still need to be addressed. The majority of actions proposed by the expert panel in 2014 were again proposed in 2017 and 2020 due to lack of progress. However, in 2020 the actions recommended were broader, reflecting a growing movement to locate nutrition within the wider context of planetary health and with recognition of the social determinants of health and nutrition. The higher-level actions proposed in 2020 will require the involvement of many government entities. It is important that Food-EPI continues to be conducted every 3 years to monitor government progress and provide a consensus view from public health experts on the most important actions required to prevent obesity and improve diets.

## Supplementary Information

**Additional file 1:** Healthy Food Environment Policy Index (Food-EPI).

**Additional file 2:** Recommended actions for the New Zealand government: Policy actions targeting food environments.

## Data Availability

The datasets used and/or analysed during the current study are available from the corresponding author on reasonable request.

## Abbreviations

Food-EPI: Healthy Food Environment Policy Index
NCD: Noncommunicable disease
