# Supplementary material for: Implementing healthy food environment policies in New Zealand: nine years of inaction
Source: Health Res Policy Syst. 2022 Jan 15;20:8. doi: 10.1186/s12961-021-00809-8 (PMC8760574; doi:10.1186/s12961-021-00809-8)
Supplement: Supplementary file 1 — Additional file 1: Healthy Food Environment Policy Index (Food-EPI). [file 12961_2021_809_MOESM1_ESM.pptx]

## Slide 1
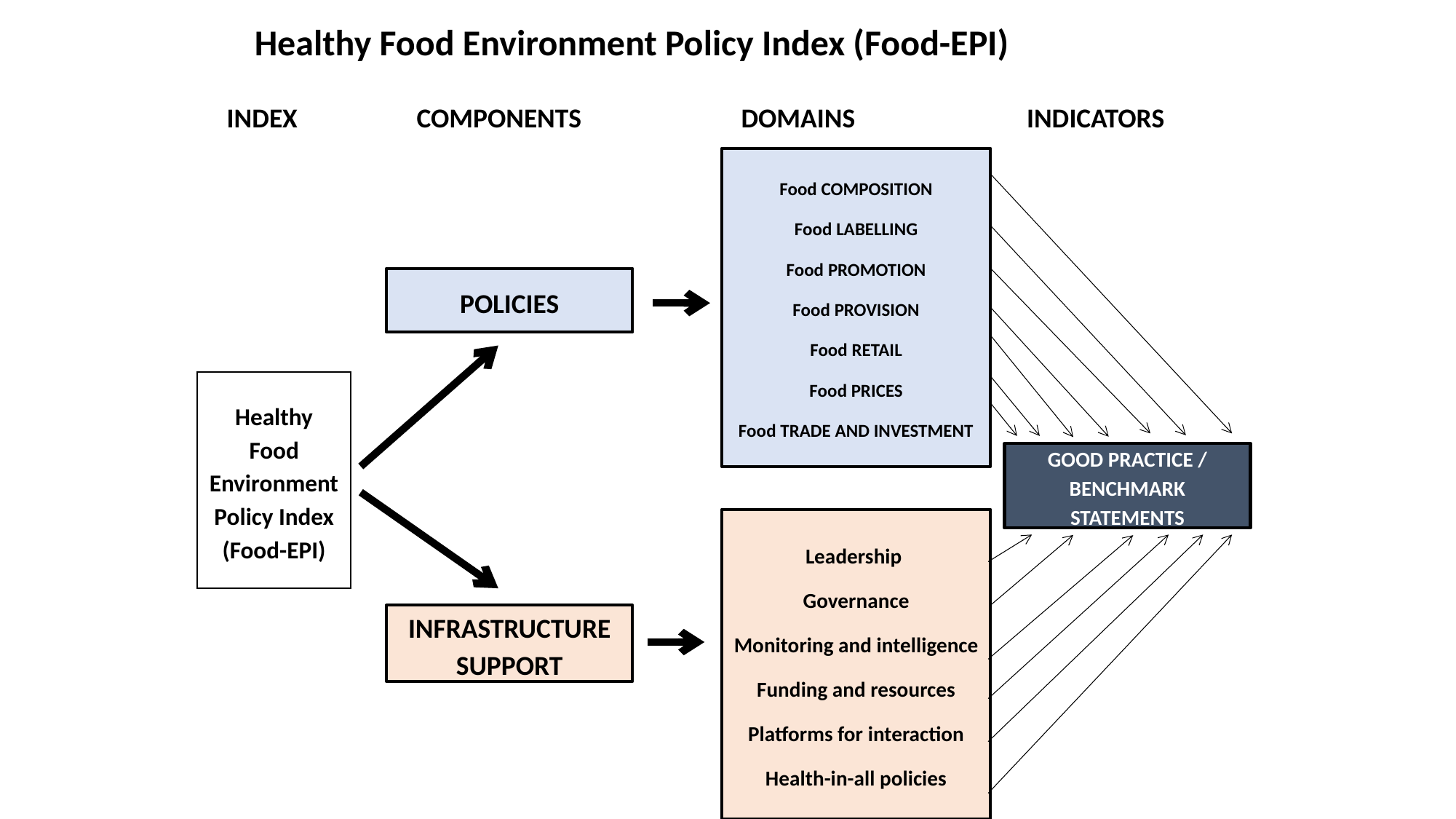

Healthy Food Environment Policy Index (Food-EPI)
INDEX
COMPONENTS
DOMAINS
INDICATORS
Food COMPOSITION
Food LABELLING
Food PROMOTION
Food PROVISION
Food RETAIL
Food PRICES
Food TRADE AND INVESTMENT
POLICIES
Healthy Food Environment Policy Index (Food-EPI)
GOOD PRACTICE / BENCHMARK STATEMENTS
Leadership
Governance
Monitoring and intelligence
Funding and resources
Platforms for interaction
Health-in-all policies
INFRASTRUCTURE SUPPORT
