# Supplementary material for: Implementing healthy food environment policies in New Zealand: nine years of inaction
Source: Health Res Policy Syst. 2022 Jan 15;20:8. doi: 10.1186/s12961-021-00809-8 (PMC8760574; doi:10.1186/s12961-021-00809-8)
Supplement: Supplementary file 2 — Additional file 2: Recommended actions for the New Zealand government: Policy actions targeting food environments. [file 12961_2021_809_MOESM2_ESM.pdf]

Additional File 2

Recommended actions for the New Zealand government: Policy actions targeting food environments

A: Higher priority recommended actions for the New Zealand government: Policy actions targeting food environments (listed in order of importance ranked by Expert Panel)

Domain	Label	Action
PROMOTION	PROMO 1<br>PROMO 2<br>PROMO 3	**Marketing to children:** The Government introduces regulations to restrict unhealthy food and beverage marketing to children up to 18 years old through broadcast media (during peak TV viewing times), non-broadcast media (including food packaging, sport sponsorship and digital media) and in children's settings, using the WHO nutrient profiling models, tailored to the NZ context.
PRICES	PRICES2	**Sugary drinks industry levy:** The Government introduces a tiered industry levy of at least 20% on sugary drinks and recycles the revenue for programmes to improve public health and well-being.
PRICES	PRICES4	**Income support:** The Government supports low income households so they can afford a healthy diet.
COMPOSITIO N	COMP1	**Food composition targets:** The Government adopts a two-tier system for reducing sodium and added sugar in key food categories: Setting mandatory maximum levels that reduce over time, and; setting and monitoring targets for voluntary reductions in sales-weighted averages.
PROVISION	PROV1	**School food policies:** The Government requires and supports schools and early childhood education services to develop food policies which ensure healthy foods are provided and promoted.
LABELING	LABEL3	**Mandatory Health Star Rating (HSR):** The Government makes the HSR mandatory and adopts the recommendations on changes to the algorithm and beverages of the 5-year review of the HSR System.
RETAIL	RETAIL 1<br>RETAIL 2	**Zoning laws:** The Government enacts zoning legislation to allow and encourage local Governments to create healthy community food environments (including school healthy food zones, community gardens and reduced concentrations of unhealthy food outlets).
PROVISION	PROV2	**Public sector healthy food:** The Government applies the healthy food and drink policy it developed for the health sector across the whole government sector.

B: Lower priority recommended actions for the New Zealand government: Policy actions targeting food environments (not ranked by experts)

Domain	Label	Action
COMPOSITION	COMP2	The Government sets a mandatory standard for deep frying oils (maximum saturated plus trans fats) for out-of-home meals and recommends targets for energy, sodium, saturated fat and sugar in Quick Service Chain Restaurant meals.
LABELING	LABEL1	The Government requires added sugars to be added on the Nutrition Information Panel
LABELING	LABEL2	The Government investigates the application of the Nutrient Profiling Scoring Criterion to restrict the use of nutrient content claims on packaged unhealthy foods (especially 'irrelevant claims' such as 'no cholesterol' claims on plant-based foods).
LABELING	LABEL4	No recommended action
PRICES	PRICES1	No recommended action
PRICES	PRICES3	The Government requires government programs that subsidize/supply food for pre-school and school-age children (or in Early Childhood Centres and schools) to meet food and nutrition guidelines.
PROVISION	PROV3	The Government increases funding for support and training of Government and children's settings to remove barriers and stimulate implementation of policies and actions to create healthy food environments at the equivalent level to the current physical activity budget.
PROVISION	PROV4	No recommended action
RETAIL	RETAIL3	The Government supports the food industry to develop SMART (Specific, Measurable, Achievable, Relevant, Time Bound) strategies and evaluate the Food Industry Taskforce commitments.
RETAIL	RETAIL4	No recommended action
TRADE	TRADE1	The Government includes formal and explicit population nutrition and health risk assessments as part of their national interest analysis on trade and investment agreements, including honouring the rights of Māori to the protection of health under the Treaty of Waitangi.
TRADE	TRADE2	The Government ensures that specific and explicit provisions are included in trade and investment agreements, allowing the New Zealand government to preserve its regulatory capacity to protect and promote public health.

C: Higher priority recommended actions for the New Zealand government: Infrastructure support actions (listed in order of importance ranked by Expert Panel)

Domain	Label	Action
LEADERSHIP	LEAD1<br>LEAD2<br>LEAD5	**National Food Strategy:** The Government develops a long-term, multi-sectoral National Food Systems and Nutrition Strategy with clear outcomes and indicators to improve sustainability, food sovereignty, health, and equity and to honour the rights of Māori to the protection of health under the Treaty of Waitangi.
MONITORING	MONIT2	**National Nutrition Survey**: The Government conducts a new national nutrition survey for children and adults to be commissioned by 2021.
LEADERSHIP	LEAD4	**Nutrition in National Strategies**: The Government ensures that the implementation plans for existing relevant Government actions such as the Child and Youth Wellbeing Strategy address the national needs and priorities to improve food environments, beyond Healthy Active Learning.
GOVERNANCE	GOVER2	**Science Input**: The government appoints a Food and Nutrition Scientific Committee to work with the Ministerial Science Advisors to ensure policies related to food and nutrition are evidence-based and equitable.
LEADERSHIP	LEAD3	**Healthy Sustainable Dietary Guidelines**: The Government actively implements and increases funding to promote Eating and Activity guidelines which incorporate the social, environmental and cultural dimensions of eating.
FUNDING	FUND1	**Nutrition Funding:** The Government increases funding for population nutrition promotion to at least 10% of obesity/overweight health care costs.
GOVERNANCE	GOVER1<br>GOVER3	**Commercial conflicts of interest**: The Government expands its conflict of interest procedures to include commercial conflicts and transparency measures so that consultation with the food industry can continue without it exerting undue influence on Government policy development.
MONITORING	MONIT1<br>MONIT6<br>HIAP1	**Monitoring Food Environments**: The Government regularly monitors the food environment for health, equity and sustainability.
MONITORING	MONIT5	**Programme Evaluation**: The Government includes robust process and impact programme evaluations in any major investment made to improve population nutrition.
PLATFORMS	PLATF1	**Cross Government Platforms for Engagement**: The Government strengthens and expands platforms for engagement for food-related prevention policies across Government (national and local).
FUNDING	FUND3	**Funding Health Promotion Authority:** The Health Promotion Agency funding is increased for promoting healthy and sustainable eating including through sector engagement towards a comprehensive approach to food policy.
PLATFORMS	PLATF4	**Community Systems Change**: The Government expands regional systems platforms (like Healthy Families New Zealand) to other regions and creates more sustainable systems platforms.
PLATFORMS	PLATF3	**Engagement Platforms with Civil Society:** The Government ensures formal platforms are created for civil society, academic and affected communities'

		input into central and local food policy development, implementation and evaluation.
FUNDING	FUND2	**Food in National Science Challenges**: The Government ensures that improving nutrition and sustainable diets and reducing nutrition inequalities is a priority funding stream within the Science Challenges and is linked to the proposed National Food and Nutrition Strategy.

D: Lower priority recommended actions for the New Zealand government: Infrastructure support actions (not ranked)

**Domain**	**Label**	**Action**
GOVERNANC E	GOVER4	The Government ensures access to comprehensive nutrition information and key documents (e.g. budget documents, annual performance reviews and health indicators) for the public.
MONITORING	MONIT3	The Government includes anthropometry monitoring, preferably within existing school-based health checks, as an indicator in the Child and Youth Wellbeing Strategy, and funds tailored whãnau support services for children with obesity.
MONITORING	MONIT4	The Government continues to invest in cardiovascular disease and diabetes risk assessments and investigates the inclusion of height and weight measurements and the use of the data for population monitoring.
PLATFORMS	PLATF2	The Government creates a meaningful, representative platform to engage and motivate industry to provide a healthier food environment.
HEALTH IN ALL POLICIES	HIAP2	The Government establishes a health impact assessment capacity, including funding for health impact assessments at the national and local level, to ensure that government policies in general are compatible with the objectives of improving health.
